# Identification of novel biomarkers and small molecule drugs in human colorectal cancer by microarray and bioinformatics analysis

**DOI:** 10.1002/mgg3.713

**Published:** 2019-05-13

**Authors:** Juan Chen, Ziheng Wang, Xianjuan Shen, Xiaopeng Cui, Yuehua Guo

**Affiliations:** ^1^ Laboratory Medicine Center People's Hospital of Hai'an County Nantong P. R. China; ^2^ Department of Clinical Biobank Nantong University Affiliated Hospital Nantong P. R. China; ^3^ Department of Medicine Nantong University Xinling college Nantong P.R. China; ^4^ Research Center of Clinical Medicine Affiliated Hospital of Nantong University Nantong P. R. China; ^5^ Department of general surgery Affiliated Hospital of Nantong University Nantong P. R. China

**Keywords:** colorectal cancer, bioinformatics, biomarker, drug

## Abstract

**Background:**

Colorectal cancer (CRC) is one of the most common malignant tumors. In the present study, the expression profile of human multistage colorectal mucosa tissues, including healthy, adenoma, and adenocarcinoma samples was downloaded to identify critical genes and potential drugs in CRC.

**Methods:**

Expression profiles, GSE33113 and GSE44076, were integrated using bioinformatics methods. Differentially expressed genes (DEGs) were analyzed by R language. Functional enrichment analyses of the DEGs were performed using the Database for Annotation, visualization, and integrated discovery (DAVID) database. Then, the search tool for the retrieval of interacting genes (STRING) database and Cytoscape were used to construct a protein–protein interaction (PPI) network and identify hub genes. Subsequently, survival analysis was performed among the key genes using Gene Expression Profiling Interactive Analysis (GEPIA). Connectivity Map (CMap) was used to query potential drugs for CRC.

**Results:**

A total of 428 upregulated genes and 751 downregulated genes in CRC were identified. The functional changes of these DEGs were mainly associated with cell cycle, oocyte meiosis, DNA replication, p53 signaling pathway, and progesterone‐mediated oocyte maturation. A PPI network was identified by STRING with 482 nodes and 2,368 edges. Survival analysis revealed that high mRNA expression of AURKA, CCNB1, CCNF, and EXO1 was significantly associated with longer overall survival. Moreover, CMap predicted a panel of small molecules as possible adjuvant drugs to treat CRC.

**Conclusion:**

Our study found key dysregulated genes involved in CRC and potential drugs to combat it, which may provide novel insights and potential biomarkers for prognosis, as well as providing new CRC treatments.

## 1 INTRODUCTION

Colorectal cancer (CRC) is one of the most common cancers with high morbidity worldwide. Despite advances in screening detection and new treatment strategies, CRC remains a leading cause of cancer‐associated mortality, due to the lack of effective diagnostic methods at the early stage and reduce sensitivity to chemotherapy (Weiser, 2018). Therefore, it is crucial to understand the precise molecular mechanisms involved in the carcinogenesis and thus develop promising prognostic biomarkers and potential therapeutic targets.

Colorectal cancer is a complex bioprocess following the adenomacarcinoma multistage sequence (Testa, Pelosi, & Castelli, 2018). Thus, molecular dysregulations during the process of carcinogenesis, particularly during the precancerous stage, should be considered as the most important risk factors contributing to the progression of CRC. Several studies have used gene expression profiling to identify key genes between CRC samples and normal samples. Huang, Yang, and Huang (2018) identified hundreds of CRC associated differentially expressed genes (DEGs) based on the Gene Expression Omnibus (GEO) and The Cancer Genome Atlas (TCGA) database. An, Zhao, Yu, and Yang (2019) found 35 genes significantly associated with patient survival based on the transcription profile of multi‐stage carcinogenesis and bioinformatics analysis. Hou et al. (2018) found a collection of DEGs and DNA methylation aberrations in CRC. Hundreds of DEGs were detected. However, DEGs are different in different studies with only some of them consistently detected. Therefore, the discovery of novel effective therapeutic targets against CRC is urgently required.

In this study, we selected the following microarray datasets GSE33113 (de Sousa et al., 2011; Kemper et al.., 2012) and GSE44076 (Closa et al., 2014; Cordero et al., 2014; Sanz‐Pamplona et al., 2014; Solé et al., 2014) from the GEO database to identify DEGs. Kyoto encyclopedia of genes and genomes (KEGG) and gene ontology (GO) pathway analysis were used to investigate DEGs. Then we identified hub genes from the common DEGs by constructing protein–protein interaction (PPI) network. Furthermore, the survival analysis was used on the gene expression profiling interactive analysis (GEPIA) website. Candidate small molecules were identified for their potential use in the treatment of CRC.

## 2 MATERIALS AND METHODS

### 2.1 Data resources

To investigate the differential gene expression between CRC and normal samples, GSE33113 and GSE44076 microarray datasets were downloaded from the GEO website (http://www.ncbi.nlm.nih.gov/geo/). These RNA profiles were provided on platform GPL570 (Affymetrix Human Genome U113 Plus 2.0 Array) (GSE33113) and GPL13667([HG‐U219] Affymetrix Human Genome U219 Array) (GSE44076). A total of 188 CRC samples and 154 normal samples were obtained in our study, including 40 tumor samples and 6 normal samples in GSE33113 profile, 98 tumor samples, and 148 normal samples in GSE44076 profile.

### 2.2 Identification of DEGs

The original CIMFast Event Language files were downloaded and classified as CRC and normal groups. The raw data were standardized and transformed into expression values using the affy package of Bioconductor (Bioconductor, http://www.bioconductor.org/). The significance analysis of the empirical bayes methods within limma package was applied to identify DEGs between CRC samples and normal tissue sample (Ritchie et al., 2015). Adj.*p* value <0.01 and |logFC| > 1 were set as the cutoff criteria to select the significant DEGs.

### 2.3 KEGG and GO enrichment analyses of DEGS

To explore potential biological process (BP), molecular functions (MF), and cellular components (CC) related to the overlap DEGs, the Database for Annotation, Visualization, and Integrated Discovery (DAVID; http://david.ncifcrf.gov) (version 6.7) was introduced to perform functional annotation and pathway enrichment analysis, including GO and KEGG pathway analysis (Ashburner et al., 2000; Dennis et al., 2003; "The Gene Ontology (GO) project," 2006; Huang, Sherman, & Lempicki, 2009; Kanehisa & Goto, 2000). GO is a major bioinformatics tool to annotate genes and analyze BP of these genes. KEGG is a database resource for understanding high‐level functions and biological systems from large‐scale molecular datasets generated by high‐throughput experimental technologies. *p* value <0.05 was considered statistically significant.

### 2.4 Protein–protein interaction (PPI) network construction and module analysis

The Search Tool for the Retrieval of Interacting Genes database (STRING, https://string-db.org/) is an online tool designed to analyze the PPI information (Damian et al., 2015). Analyzing the functional interactions between proteins may provide insights into the mechanisms of generation or development of diseases. In the present study, all the previously identified DEGs were submitted to STRING database for exploring their potential interactions. The interactions with a combined score >0.4 were considered significant and extracted for constructing the PPI networks through the Cytoscape software that is an open source bioinformatics software platform for visualizing molecular interaction networks (Bandettini et al., 2012). Subsequently, Molecular Complex Detection (MCODE) was used to screen significant modules from the PPI network with degree cutoff = 2, node score cutoff = 0.2, k‐core = 2, and max depth = 100 (Smoot, Ono, Ruscheinski, Wang, Ono, Ruscheinski, Wang, & Ideker, 2011). The functional and pathway enrichment analysis of for the significant modules was also performed. The networks gene oncology tool (BiNGO) plugin of Cytoscape was used to perform and visualize the BP analysis of the hub genes (Maere, Heymans, & Kuiper, 2005).

### 2.5 Analysis and validation of hub genes

A network of module genes and their co‐expression genes was established by cBioPortal online platform (http://www.cbioportal.org). To confirm the reliability of hub genes from our detection, we analyzed their prognostic and expression in CRC using Gene Expression Profiling Interactive Analysis (GEPIA), an interactive web application tool for gene expression analysis, containing 8,587 normal samples and 9,736 tumors samples from the Genotype‐Tissue Expression databases and TCGA databases (Cerami et al., 2012; Gao et al., 2013; Tang et al., 2017). Then the survival curve and box plot were performed to visualize the relationships. In addition, the protein expression of the hub genes between CRC and normal tissues was determined by the human protein atlas (HPA, www.proteinatlas.org) database, an online tool for analyzing protein level from clinical samples.

### 2.6 Identification of small molecules

The Connectivity Map (CMap, http://www.broadinstitute.org/cmap/) was used to predict potential small active molecular that may induce or reverse the current biological status encoded by a particular gene expression signature (Lamb et al., 2006). We contrasted the DEGs with those participating in small active molecular interference in the CMap database to find potential small molecular related to these DEGs. First, the overlaps DEGs were divided into upregulated and downregulated groups. Then, these probe sets were used to query the CMap database. Finally, the enrichment score representing similarity was calculated, ranging from −1 to 1. A positive connectivity value (closer to +1) indicated the small molecules could induce the state of CRC cells, whereas a negative connectivity value (closer to −1) indicated greater similarity among the genes and the small molecules could reverse the above cancer cell status.

## 3 RESULTS

### 3.1 Identification of DEGs in CRC

**Figure 1:**
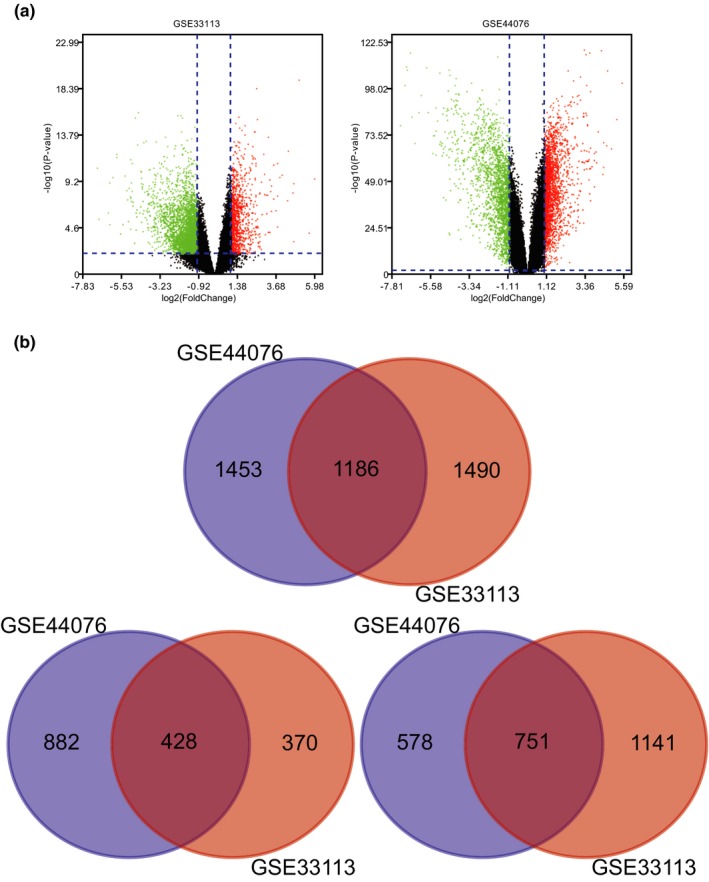
(a) Volcano plot of gene expression profile data between CRC and normal tissues in each dataset. Red dots: significantly upregulated genes in CRC; Green dots: significantly downregulated genes in CRC; Black dots: nondifferentially expressed genes. Adj. *p* < 0.01 and |log2 FC| > 1 were considered as significant. (b) A. Venn diagram of 1,186 overlapping DEGs from GSE33113 and GSE44076 datasets including 428 upregulated DEGs and 751 downregulated DEGs. CRC, Colorectal cancer; DEGs, Differentially expressed genes

Analyzed with the Limma package, a total of 1,186 overlap DEGs expressed in CRC samples were extracted from the GSE33113 and GSE44076 datasets. The volcano plot of DEGs of CRC in each dataset was presented in Figure 1a. The Venn diagrams showed the 1,186 overlap DEGs among the three datasets (Figure 1bA) including 428 significantly upregulated genes (Figure 1bB) and 751 downregulated genes (Figure 1bC).

### 3.2 Enrichment analyses

**Figure 2:**
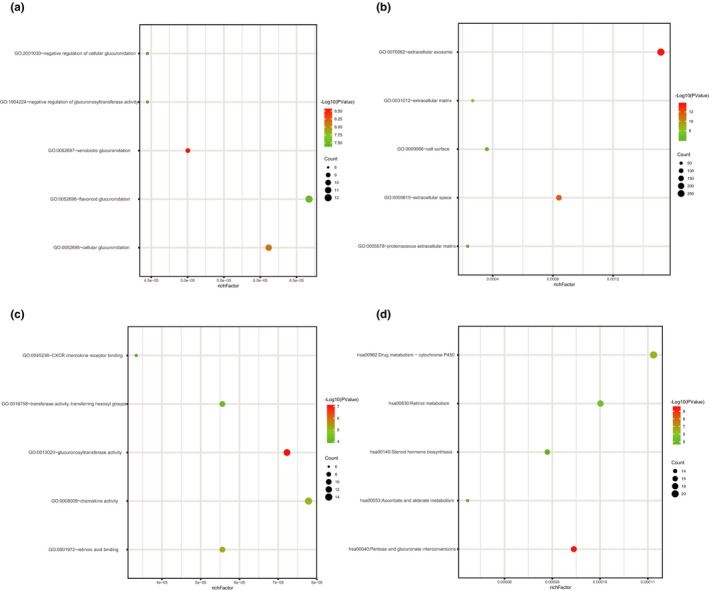
Functional and signaling pathway analysis of the overlapped DEGs in CRC. (a) Biological processes (b) Cellular components (c) Molecular function (d) KEGG pathway. CRC, Colorectal cancer; DEGs, Differentially expressed genes; KEGG, Kyoto Encyclopedia of Genes and Genomes

To explore the biological functions of identified DEGs, we performed functional and pathway enrichment analyses using DAVID. For biological processes, GO analysis results indicated that upregulated and downregulated DEGs were significantly enriched in xenobiotic glucuronidation, cellular glucuronidation, flavonoid glucuronidation, negative regulation of cellular glucuronidation, and negative regulation of glucuronosyl transferase activity. Cell component analysis showed that these DEGs were particularly involved in extracellular exosome, extracellular space, extracellular matrix, proteinaceous extracellular matrix, and cell surface. Similarly, changes in molecular function of DEGs were significantly enriched in glucuronosyl transferase activity, retinoic acid binding, chemokine activity, transferase activity, transferring hexosyl groups, and CXCR chemokine receptor binding. Additionally, the results of KEGG pathway analysis revealed that these DEGs were mainly enriched in Pentose and glucuronate interconversions, Ascorbate and aldarate metabolism, Drug metabolism‐cytochrome P450, Retinol metabolism, and Steroid hormone biosynthesis (Figure 2 & Table 1).

**Table 1:** Functional and pathway enrichment analysis of the overlap DEGs

Category	Term	*p* Value	Genes
GOTERM_BP_DIRECT	GO:0052697~xenobiotic glucuronidation	2.85E‐09	UGT1A7, UGT1A10, UGT1A6, UGT1A9, UGT1A8, UGT1A3, UGT1A5, UGT1A4, UGT1A1
GOTERM_BP_DIRECT	GO:0052695~cellular glucuronidation	7.93E‐09	UGT1A7, UGT1A10, UGT1A6, UGT1A9, UGT2B17, UGT1A8, UGT1A3, UGT1A4, UGT2A3, UGT2B15, UGT1A1
GOTERM_BP_DIRECT	GO:0052696~flavonoid glucuronidation	3.33E‐08	UGT1A7, UGT1A10, UGT1A6, UGT1A9, UGT2B17, UGT1A8, UGT1A3, UGT1A5, UGT1A4, UGT2A3, UGT2B15, UGT1A1
GOTERM_BP_DIRECT	GO:2001030~negative regulation of cellular glucuronidation	3.88E‐08	UGT1A7, UGT1A10, UGT1A6, UGT1A9, UGT1A8, UGT1A3, UGT1A4, UGT1A1
GOTERM_BP_DIRECT	GO:1904224~negative regulation of glucuronosyltransferase activity	3.88E‐08	UGT1A7, UGT1A10, UGT1A6, UGT1A9, UGT1A8, UGT1A3, UGT1A4, UGT1A1
GOTERM_CC_DIRECT	GO:0070062~extracellular exosome	2.01E‐14	LTBP4, PTGS1, CCT2, SELENBP1, EDIL3, OGN, ACTG2, CD44, CD46, SERPINE1, CFH, COL12A1, GNG2, CFI, GUCA2A, CFD, GUCA2B, RAB27A, GNG7, ACAA2, BSG, CLCA4, CRYAB, RAN, CDHR2, KRT12, LIFR, CDHR5, COLEC12, GLTP, THY1, PRELP, NAPEPLD, TACSTD2, PGM1, RAB15, GCNT3, AHCY, GNAI1, MME, SERPINH1, EPHB4, ACAT1, DAAM2, KRT24, GPD1L, SERINC2, SIAE, FGL2, FAM162A, SPP1, BMP3, MGAT4A, CFB, MAOB, IL1RN, GARS, S100A11, C16ORF89, HBA2, HBA1, LGALS9, GART, PPA1, GGT6, CD55, CKMT1A, SFRP1, FCGBP, PLAU, CKMT1B, NAAA, ANPEP, SLC26A2, SPINK5, CKB, HSPH1, SLC23A1, SMPDL3B, SMPDL3A, GPX3, SLC22A5, FAM129A, CEACAM1, KCNMA1, CCT6A, FLNB, TRAP1, MTMR11, PFDN2, CHMP1B, BGN, SERPINB5, CLIC5, CLIC6, RUVBL1, CHL1, C7, SORD, UGDH, CLDN11, ITM2C, CCL28, ITM2A, GLIPR2, GPM6A, CD177, AGT, SULT1C2, C2, THBS1, CD27, TMPRSS2, CES3, BCAS1, CES2, SHMT2, TRHDE, CAPN5, SI, APPL2, PCK1, FBLN1, PROM2, GLA, PLSCR4, TOM1L2, PTP4A1, EEF1E1, GFRA1, CP, PSAT1, PAICS, HPGD, CDH11, GNPDA1, TSPAN1, NIT1, THRB, TSPAN3, MMP9, GNA11, IQGAP2, SLC7A5, CXCL12, CMBL, PRKAR2B, ASPA, SLC16A1, TGFBI, IL1B, PRKACB, SLC4A4, NEGR1, MB, COCH, SPARCL1, RBKS, METTL7A, NEBL, PCOLCE2, TRIM36, AKR1B10, COL1A2, SNRPB, MFAP4, DST, SRI, WNT5A, FXYD3, PPIL1, ITLN1, CLU, PSMA7, TIMP1, B3GNT8, REG1A, CSE1L, OSBPL1A, ENTPD5, ANGPTL1, NDRG2, DEFB1, FN1, CPNE8, S100P, CPNE5, MYO1D, EPHX2, SLC6A14, SPPL2A, ATAD2, ABCB1, ENDOD1, SLC6A19, MUC4, LCN2, TST, LYVE1, PLCG2, MYH11, FABP4, SLC13A2, HSPD1, ANTXR1, MYLK, PRPH, FIGNL1, SLC5A1, IGFBP6, CLDN5, PRDX4, FAM63A, AMN, DES, GSN, ACOT11, PDE8A, RHOF, AHNAK, AKR1C1, CDK1, ACADM, CILP, MPP6, PADI2, PIGR, MAN1A1, PRKCB, CFL2, SCIN, CA4, WASL, CA2, CA1, CTSG, UGP2, CPM, ADH6, OAF, PBLD, UGT1A6, MTM1, UGT1A9, HSPA2, ATIC, PAFAH1B3, PLCD1, SCNN1G, NEDD4L, SLC39A5, SCNN1B, COL8A1, HBB, MYOC, DPT, GSTA1, SECTM1, RNASE1, TNXB, OLR1, TNXA, SLC12A2, KL, NFASC, KLK1, GDPD3, FUCA1, ANXA3, SMPD1, DSC2, GDF15, ATP8A1
GOTERM_CC_DIRECT	GO:0005615~extracellular space	2.08E‐12	EDN3, CTHRC1, RETNLB, MMP9, LTBP4, SELENBP1, MMP3, CXCL11, CXCL12, CXCL10, REG3A, ACTG2, OGN, SERPINE2, SOSTDC1, FAP, TGFBI, SERPINE1, CFH, COL12A1, IL1B, CFI, CFD, LGI1, MTUS1, SPON1, SPARCL1, C10ORF99, PRELP, SCGB2A1, TNFAIP6, ADAMTS9, CD36, TACSTD2, VEGFA, COL1A2, STC1, RELN, COL1A1, WNT5A, ODAM, ENPP2, CLU, CCL8, FAM132A, CHEK1, GREM2, SERPINH1, ABI3BP, TIMP1, SIAE, ANGPTL1, DEFB1, OLFM1, SPP1, FN1, BMP3, BMP2, CFB, LGALS4, IL1RN, S100A11, LMCD1, TINAG, LGALS9, MUC4, C2ORF40, TST, LCN2, LAMA1, TNFSF11, NPY, SRPX2, S100B, SFRP1, SFRP4, NLGN4X, POP1, HSPD1, PYY, AREG, TPSAB1, SST, PLAU, IGFBP6, PRDX4, SPINK2, ANPEP, AMN, CKB, MTHFD2, SMPDL3B, GSN, SMPDL3A, GPX3, SEMA3D, LOXL2, ANGPT2, GHR, EGFL6, CILP, PIGR, IL6R, SLIT3, CHGA, SEMA4G, SERPINB5, CFL2, VCAN, CA2, CTSG, CXCL1, CPM, SORD, CXCL5, VPREB3, TNC, CXCL3, CXCL2, CXCL9, CXCL6, KIT, CCL28, PCSK2, CCL23, CCL20, AGT, PTN, PCSK9, C2, THBS1, MYOC, SCG2, DPT, CES3, SECTM1, CES2, TNXB, TNFSF4, KL, SPARC, GCG, DKK2, FBLN1, CCL13, SULF1, SMPD1, CMTM7, MEP1B, CP, GDF15
GOTERM_CC_DIRECT	GO:0031012~extracellular matrix	3.99E‐09	FGFR2, LTBP4, TNC, CLU, CCT2, EDIL3, MMRN1, ABI3BP, MMP1, OGN, SERPINE2, TGFBI, SERPINE1, COL12A1, COL8A1, LOXL2, THBS1, THBS2, MYOC, DPT, SPON1, FN1, COCH, HAPLN1, TNXB, COL4A1, RAN, CILP, LMCD1, CCT6A, MMP14, COL5A2, FLNB, MMP11, PRELP, LAMA1, ADAMTS9, FBLN1, BGN, SFRP1, ZG16, COL1A2, VCAN, COL1A1, HSPD1, TPSAB1, MFAP4, CTSG
GOTERM_CC_DIRECT	GO:0005578~proteinaceous extracellular matrix	1.12E‐07	WNT5A, CTHRC1, MAMDC2, MMP9, LTBP4, MMP28, MMP3, MMP1, TIMP1, OGN, TGFBI, MYOC, DPT, SPON1, FN1, COCH, HAPLN1, TNXB, TNXA, SPARCL1, OLFML2B, CILP, SPARC, COL5A2, COL4A6, MMP12, MUC4, SLIT3, MMP11, COL4A5, PRELP, LAMA1, ADAMTS9, FBLN1, BGN, SFRP1, ZG16, VEGFA, COL1A2, VCAN, RELN, CHL1
GOTERM_CC_DIRECT	GO:0009986~cell surface	8.51E‐07	TLR2, TLR3, IQGAP2, LPAR1, SRPX, CD44, ANK3, CD46, FAP, RSPO2, CHRNA1, CEACAM1, GHR, TMEM206, CRYAB, NRXN1, IL6R, SLC7A11, ADAMTS9, BGN, CD36, TNFRSF10B, BACE2, VEGFA, CA4, SLITRK6, CTSG, AOC3, WNT5A, FGFR2, CPM, FGFR3, TNFRSF12A, CLU, HMMR, SLC11A2, FOLR2, HSPA2, P2RY1, PCSK9, PTN, SLC39A6, THBS1, FCER1A, BMP2, CAPN5, TNFSF4, MET, ITGA2, ABCB1, SPARC, FZD5, CD1D, CD55, PROM2, SRPX2, SFRP1, NLGN4X, SULF1, SFRP4, AREG, ANTXR1, HSPD1, SCARA5, PLAU
GOTERM_MF_DIRECT	GO:0015020~glucuronosyltransferase activity	8.82E‐08	CSGALNACT1, UGT1A7, UGT1A10, UGT1A6, UGT1A9, UGT2B17, UGT1A8, UGT1A3, UGT1A5, UGT1A4, UGT2A3, UGT2B15, UGT1A1
GOTERM_MF_DIRECT	GO:0001972~retinoic acid binding	6.55E‐06	UGT1A7, UGT1A10, UGT1A6, UGT1A9, UGT2B17, UGT1A8, UGT1A3, UGT1A4, UGT2B15, UGT1A1
GOTERM_MF_DIRECT	GO:0008009~chemokine activity	8.97E‐06	CXCL1, CCL13, CCL23, CXCL5, CCL20, CXCL3, CXCL2, CXCL9, CCL8, CXCL6, CXCL11, CXCL12, CCL28, CXCL10
GOTERM_MF_DIRECT	GO:0016758~transferase activity transferring hexosyl groups	7.61E‐05	UGT1A7, UGT1A6, MGAT4A, UGT1A9, UGT1A8, UGT1A3, UGT1A4, UGT2A3, UGT2B15, UGT1A1
GOTERM_MF_DIRECT	GO:0045236~CXCR chemokine receptor binding	1.11E‐04	CXCL1, CXCL5, CXCL3, CXCL2, CXCL6, CXCL12
KEGG_PATHWAY	hsa00040:Pentose and glucuronate interconversions	3.08E‐10	SORD, KL, UGDH, UGT1A1, UGT1A7, UGT1A10, UGT1A6, UGT1A9, UGT2B17, UGT1A8, UGT1A3, AKR1B10, UGT1A5, UGT1A4, UGT2A3, UGT2B15, UGP2
KEGG_PATHWAY	hsa00053:Ascorbate and aldarate metabolism	1.83E‐07	UGT1A7, UGT1A10, UGT1A6, UGT1A9, UGT2B17, UGT1A8, UGT1A3, UGT1A5, UGT1A4, UGDH, UGT2A3, UGT2B15, UGT1A1
KEGG_PATHWAY	hsa00982:Drug metabolism ‐ cytochrome P450	3.48E‐07	GSTA1, MAOA, MAOB, ADH1C, ADH1B, ADH6, UGT1A1, FMO4, UGT1A7, UGT1A10, UGT1A6, FMO5, UGT1A9, UGT2B17, UGT1A8, UGT1A3, UGT1A5, UGT1A4, UGT2A3, UGT2B15
KEGG_PATHWAY	hsa00830:Retinol metabolism	3.13E‐06	ADH1C, ADH1B, ADH6, DHRS9, UGT1A1, RDH5, UGT1A7, UGT1A6, UGT1A10, UGT1A9, UGT2B17, UGT1A8, UGT1A3, UGT1A5, UGT1A4, UGT2A3, UGT2B15, RETSAT
KEGG_PATHWAY	hsa00140:Steroid hormone biosynthesis	1.70E‐‐05	HSD3B2, HSD17B2, UGT1A1, UGT1A7, UGT1A6, UGT1A10, UGT1A9, UGT2B17, UGT1A8, UGT1A3, UGT1A5, UGT1A4, HSD11B2, UGT2A3, UGT2B15, AKR1C1

Abbreviations: CRC, Colorectal cancer; DEGs, Differentially expressed genes

### 3.3 PPI network construction and module analysis

**Figure 3:**
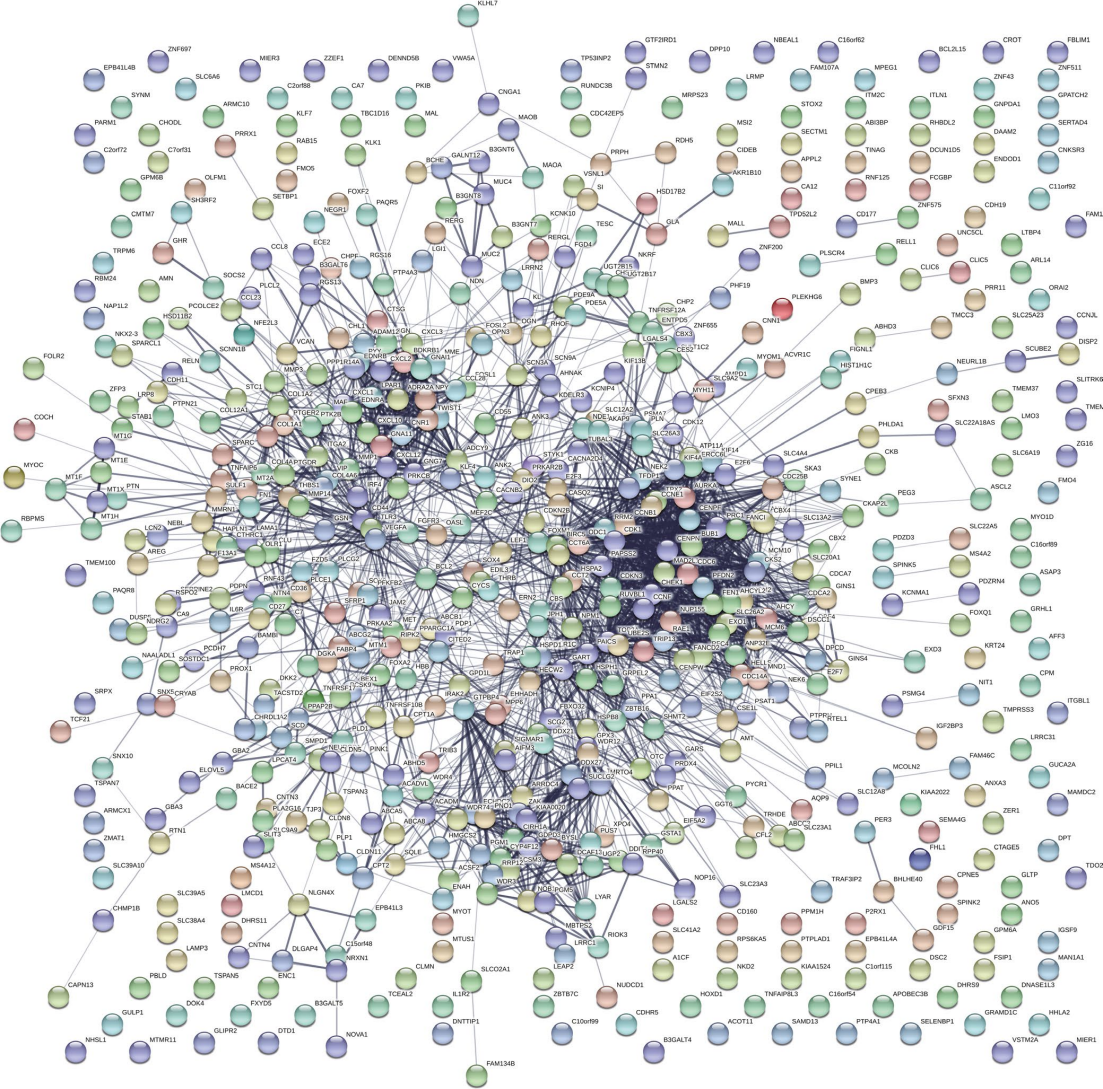
Protein–protein interaction networks construction

**Figure 4:**
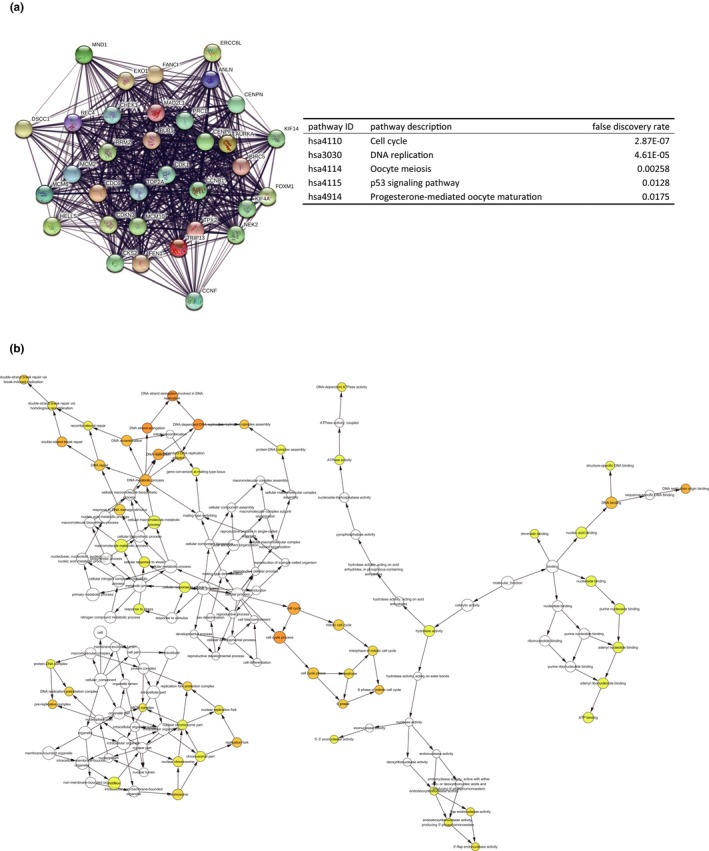
(a)The top three most significant modules extracted from PPI network and KEGG pathway analysis of module genes. (b)The biological process of module genes analyzed by BiNGO. The color depth of nodes represents the corrected *p*‐value. The size of nodes represents the number of genes involved. PPI, protein–protein interaction; KEGG, Kyoto Encyclopedia of Genes and Genomes

Based on the STRING database, a PPI network of DEGs with 482 nodes and 2,368 interactions was established using the Cytoscape software (Figure 3). The most significant modules were extracted from this PPI network by MCODE (Figure 4a). The results of signaling pathway enrichment analysis suggested that the module genes were mainly enriched in cell cycle, DNA replication, oocyte meiosis, p53 signaling pathway, and progesterone‐mediated oocyte maturation (Table 2). The BP analysis showed that the module genes were significantly related to DNA replication, DNA strand elongation, and DNA‐dependent DNA replication (Figure 4b)

**Table 2:** The pathway enrichment analysis of module genes

pathway ID	pathway description	false discovery rate
hsa4110	Cell cycle	2.87E‐07
hsa3030	DNA replication	4.61E‐05
hsa4114	Oocyte meiosis	0.00258
hsa4115	*p*53 signaling pathway	0.0128
hsa4914	Progesterone‐mediated oocyte maturation	0.0175

### 3.4 Analysis and validation of hub genes

**Figure 5:**
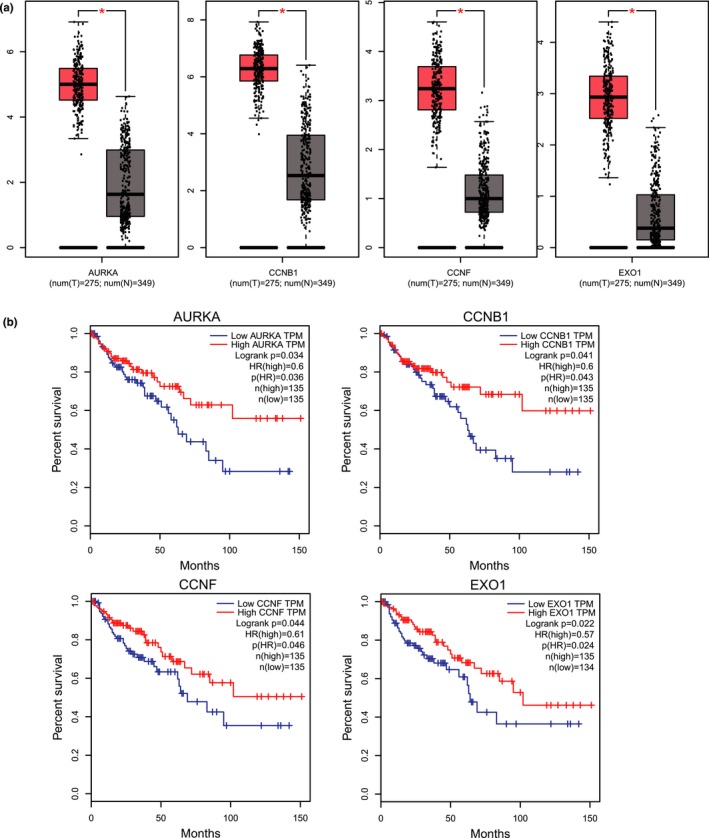
The expression level and prognostic value of hub genes according to the GEPIA database. GEPIA, Gene Expression Profiling Interactive Analysis

**Figure 6:**
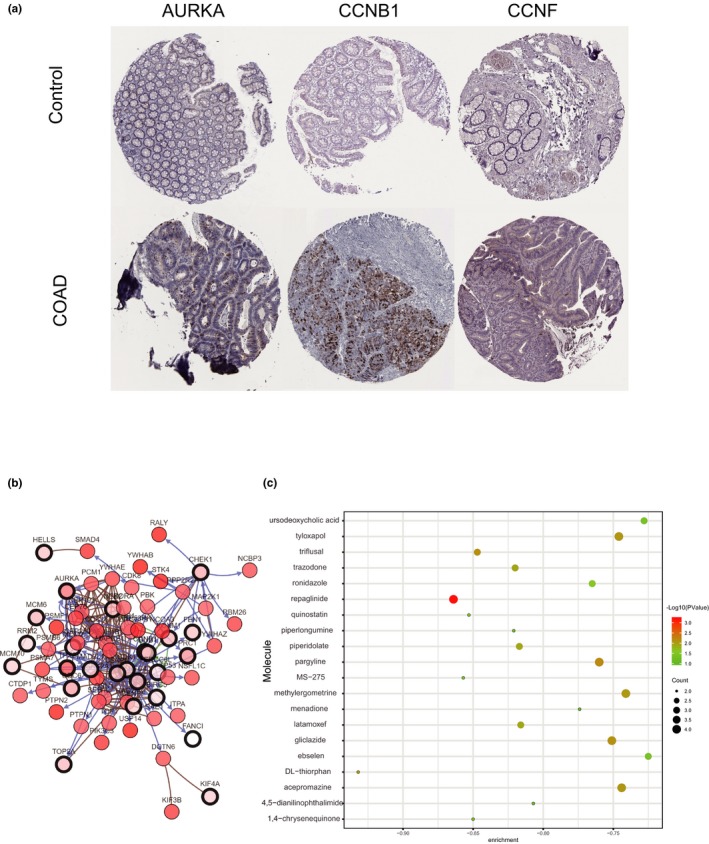
(a) Representative immunohistochemistry staining results reveal the protein level expression of hub genes in CRC and normal tissues. (b) The network of module genes and their co‐expression genes constructed by cBioPortal. Nodes with thick outline: hub genes; Nodes with thin outline: co‐expression genes. (c) Pop plot of top 20 identified small molecules that could reverse the gene expression of CRC. CRC, Colorectal cancer

In order to validate the correlation between hub genes expression and the clinical characteristics of CRC, HPA database, and the cBioPortal for Cancer Genomics database were used for further analysis. The mining of GEPIA database also demonstrated that DEGs exhibited significant differences in expression between CRC and normal tissues. This result further confirmed that the expression level of these hub genes was closely correlated with the onset of CRC. A total of 270 CRC patients were available from GEPIA database for the overall survival analysis and divided into high expression and low expression groups. It was found that all the four hub genes were upregulated (Figure 5a). Together, the expression level of AURKA, CCNB1, CCNF, and EXO1 could represent the important prognostic biomarkers for predicting the survival of CRC patients (Figure 5b). AURKA, CCNB1, CCNF, and EXO1 were selected as hub genes for further analysis. The full names and function roles for these hub genes were shown in Table 3. The immunohistochemical staining results from HPA database indicated significantly higher positivity for AURKA, CCNB1, CCNF, and EXO1(Not found in HPA database) in cancer tissues than in adjacent normal tissues (Figure 6a). A network of the module genes and their co‐expression genes was constructed using cBioPortal online platform (Figure 6b).

**Table 3:** The full name, functional roles, *p*‐value, and LogFC of hub genes

gene symbol	Summary	GSE33113	GSE44076
AURKA	The protein encoded by this gene is a cell cycle‐regulated kinase that appears to be involved in microtubule formation and/or stabilization at the spindle pole during chromosome segregation. The encoded protein is found at the centrosome in interphase cells and at the spindle poles in mitosis. This gene may play a role in tumor development and progression. A processed pseudogene of this gene has been found on chromosome 1, and an unprocessed pseudogene has been found on chromosome 10. Multiple transcript variants encoding the same protein have been found for this gene.	*p *value = 3.87e‐07, LogFC = 1.60	*p* Value = 4.11e‐51, LogFC = 1.65
CCNB1	The protein encoded by this gene is a regulatory protein involved in mitosis. The gene product complexes with p34(cdc2) to form the maturation‐promoting factor (MPF). The encoded protein is necessary for proper control of the G2/M transition phase of the cell cycle.	*p* Value = 2.41e−05, LogFC = 1.31	*p* Value = 3.56e−37, LogFC = 1.71
CCNF	This gene encodes a member of the cyclin family. Cyclins are important regulators of cell cycle transitions through their ability to bind and activate cyclin‐dependent protein kinases. This member also belongs to the F‐box protein family which is characterized by an approximately 40 amino acid motif, the F‐box. The F‐box proteins constitute one of the four subunits of the ubiquitin protein ligase complex called SCFs (SKP1‐cullin‐F‐box), which function in phosphorylation‐dependent ubiquitination. The F‐box proteins are divided into three classes: Fbws containing WD−40 domains, Fbls containing leucine‐rich repeats, and Fbxs containing either different protein–protein interaction modules or no recognizable motifs. The protein encoded by this gene belongs to the Fbxs class and it was one of the first proteins in which the F‐box motif was identified.	*p* Value = 1.80e‐06, LogFC = 1.03	*p* Value = 2.58e‐44, LogFC = 1.32
EXO1	This gene encodes a protein with 5' to 3' exonuclease activity as well as an RNase H activity. It is similar to the Saccharomyces cerevisiae protein Exo1 which interacts with Msh2 and which is involved in mismatch repair and recombination. Alternative splicing of this gene results in three transcript variants encoding two different isoforms.	*p* value = 4.39e‐10, LogFC = 2.43	*p* value = 1.20e‐43, LogFC = 1.23

### 3.5 Identification of related active small molecules

To screen and identify candidate small molecules for potential therapeutic drugs in CRC, we uploaded upregulated and downregulated DEGs groups into the CMap database for Gene Set Enrichment Analysis and then matched them with small molecule treatment. This procedure aimed to find some small molecules that could reverse the changes of gene expression in CRC. Table 4 and Figure 6c listed the top 15 most significant small molecules with their enrichment scores and p value. Among these molecules, DL‐thiorphan, repaglinide, MS‐275, and quinostatin showed higher negative correlation and the potential to treat CRC.

**Table 4:** List of the top 15 most significant small molecule drugs that could reverse the tumoral status of colorectal cancer (CRC)

cmap name	enrichment	*p*
DL‐thiorphan	−0.932	0.00972
repaglinide	−0.864	0.00062
MS–275	−0.857	0.04066
quinostatin	−0.853	0.04298
1,4‐chrysenequinone	−0.85	0.04519
triflusal	−0.847	0.00717
trazodone	−0.82	0.0117
piperidolate	−0.817	0.0121
latamoxef	−0.816	0.01232
ronidazole	−0.765	0.02646
pargyline	−0.76	0.00676
gliclazide	−0.751	0.00774
tyloxapol	−0.746	0.00824
acepromazine	−0.744	0.00851
methylergometrine	−0.741	0.00881

## 4 DISCUSSION

Although many studies on CRC development are available, more efforts are needed to identify driver genes and candidate drugs. This study integrated two gene profile datasets and utilized bioinformatics methods to analyze these datasets, and identified 1,179 commonly changed DEGs (428 upregulated and 751 downregulated). Pathway enrichment analysis indicated that that the module genes were mainly enriched in cell cycle, DNA replication, oocyte meiosis, p53 signaling pathway, and progesterone‐mediated oocyte maturation. The PPI network was constructed including 482 nodes and 2,368 edges. AURKA, CCNB1, CCNF, and EXO1 were clearly related to the prognosis of patients. In addition, small molecules that can provide new insights in CRC therapeutic studies were identified.

Many researchers have found that four key genes were involved in cell cycle, participating in tumorigenesis and tumor proliferation. AURKA has been studied in a wide range of human malignancies and is associated with poor prognosis in several malignancies, including cervical squamous cell carcinoma, hepatocellular carcinoma, and nonsmall cell lung carcinoma (Ma et al., 2017; Wang et al., 2018; Zheng et al., 2018). Besides, Yang et al., (2017) reported that AURKA as a transactivating co‐factor in the induction of the c‐Myc oncoprotein in breast cancer stem cells (BCSCs). In CRC, AURKA is critical for chromosome 20q amplification‐associated malignant transformation in colorectal adenomas (Chuang et al., 2016). CCNB1 acts as a central protein of cell cycle. Owing to its function, it is found that CCNB1 is generally abnormal in tumors. CCNB1 was associated with pathologic grade and metastasis of tumors in cases of human breast and ovarian cancer (Fei et al., 2018). Several literatures found that the expression of CCNB1 was significantly associated with pathologic grade, metastasis, and prognosis of tumors (Ding et al., 2018; Zuryn, Krajewski, Klimaszewska‐Wisniewska, Grzanka, & Grzanka, 2019). CCNF, capable of forming Skp1‐Cul1‐F‐box protein ubiquitin ligase complex, is implicated in controlling centrosome duplication and preventing genome instability. Gagat, Krajewski, Grzanka, and Grzanka (2018) revealed that high expression of CCNF in melanoma patients was associated with worse overall survival. Additionally, with gene network reconstruction, CCNF was regarded as one of the main drivers in cell cycle network in gastric cancer (Zhao et al., 2019). But Fu et al. (2013) found that CCNF was downregulated in HCC, which was an independent poor prognostic marker for overall survival. However, the function of CCNF in CRC is not clear.

The contribution of EXO1 in the safeguarding stability of the genome during DNA replicative and postreplicative processes is well‐established. EXO1 activity contributes to several DNA repair processes. EXO1 has been associated with different types of cancers owning to its mutations, including colon, breast, ovarian, lung, pancreatic, and gastric tract cancer (Bau et al., 2009; Hansen et al., 2015; Jin et al., 2008; Sun, Zheng, & Shen, 2002). Nevertheless, the overexpression of EXO1 has also been reported in several other cancers associated with poor prognosis, which in part is related to increased DNA repair activity (Axelsen, Lotem, Sachs, & Domany, 2007; Dai et al., 2018; de Sousa et al., 2017; Muthuswami et al., 2013). In this study, AURKA, CCNB1, CCNF, and EXO1 were significantly upregulated in CRC compared with normal samples, and in CRC patients, the survival rate was positively correlated with the high expression of these genes.

Several small molecules with potential therapeutic efficacy against CRC were identified. The most significant small molecules in our result have been reported to display anticancer activity. DL‐thiorphan is served as the specific neutral endopeptidase (NEP) inhibitor, which is widely used to differentiate NEP enzyme activity. NEP enzyme is a membrane‐bound metallopeptidase that plays key roles in wound repair (Muangman, Tamura, & Gibran, 2005). DL‐thiorphan may be the target and candidate agent for Type 2 Diabetes Treatment (Wang, Zhao, Shang, & Xia, 2014). Besides, thiorphan binding to CD10 might interfere with the processing of neuropeptide hemoregulatory factors and thus influence the progenitor cell proliferation in acute leukemia (Feng et al., 2011). However, there are insufficient evidences indicating DL‐thiorphan can be directly used in anticancer. Moreover, repaglininde is a new class of oral antidiabetic agents, which stimulates insulin release within a few minutes by inhibiting ATP‐sensitive potassium channels of the beta‐cell membrane via binding to a receptor distinct from that of sulphonylureas. A previous study revealed that repaglininde may have direct antitumor effects and have been shown to suppress various types of cancer cells in cell culture and in animal models (Stanovic et al., 2000). On the other hand, El Sharkawi et al reported that repaglininde may have cytotoxic effects against hepatic, breast, and cervical carcinoma cells (El Sharkawi, Shemy, & Khaled, 2014). Thus, we might suppose that these identified drugs could play certain roles to combat CRC. However, further studies were still required to clarify the role of these candidate small molecules in the pathogenesis of CRC.

## 5 CONCLUSION

Using bioinformatics analysis, 1,179 DEGs were identified, which were significantly enriched in several pathways, mainly associated with cell cycle, oocyte meiosis, DNA replication, p53 signaling pathway, and progesterone‐mediated oocyte maturation. We also identified key genes including AURKA, CCNB1, CCNF, and EXO1 that might play important roles in CRC and that might represent novel biomarkers in CRC prognosis and therapy. Additionally, a group of small molecules was identified that might be exploited as adjuvant drugs for improved therapeutics for CRC. However, further investigations are required to validate the predicted drugs.

## CONFLICT OF INTEREST

The authors report no conflict of interest in this work.
